# First Evidence of Mineralocorticoid Receptor Gene and Protein Expression in Rat and Human Thyroid Tissues and Cell Cultures

**DOI:** 10.3390/ijms25020754

**Published:** 2024-01-06

**Authors:** Jacopo Manso, Maria Chiara Pedron, Alberto Mondin, Simona Censi, Gianmaria Pennelli, Francesca Galuppini, Susi Barollo, Loris Bertazza, Claudia Maria Radu, Francesca Ghini, Paolo Simioni, Chiara Sabbadin, Filippo Ceccato, Decio Armanini, Caterina Mian

**Affiliations:** 1 Endocrinology Unit, Department of Medicine (DIMED), University of Padua, Via Ospedale Civile 105, 35128 Padua, Italy; jacopo.manso@gmail.com (J.M.);; 2 Surgical Pathology Unit, Department of Medicine (DIMED), University of Padua, Via Ospedale Civile 105, 35128 Padua, Italy; 3 General Internal Medicine and Thrombotic and Haemorrhagic Diseases Unit, Department of Medicine (DIMED), University of Padua, Via Ospedale Civile 105, 35128 Padua, Italy

**Keywords:** thyroid, aldosterone, mineralocorticoid, thyroid cancer, papillary thyroid cancer, mineralocorticoid receptor

## Abstract

Aldosterone (Aldo) exerts its action through binding with the mineralocorticoid receptor (MR). Clinically, a link between primary aldosteronism (PA) and thyroid diseases has been hypothesised. However, the presence and activity of MR on the thyroid have not yet been demonstrated. We investigated the gene/protein expression and activation of MR in primary thyroid cell cultures (normal rat thyroid [FRTL-5] and human papillary thyroid cancer [PTC] cell lines, BCPAP and K1) through qRT-PCR analysis, immunofluorescence, and confocal microscopy. We also studied the effects of Aldo on thyroid-specific and inflammation genes in vitro. Paired human normal and neoplastic thyroid tissues were also studied. We demonstrated both gene and protein expression and activation of MR in normal rat thyroid and human PTC lines. Incubation with Aldo induced an acute increase in IL-6 expression in both the FRTL-5 and BCPAP lines, which was antagonised by spironolactone, and an acute and late upregulation of thyroid-specific genes in FRTL-5. MR was also expressed at both gene and protein levels in normal human thyroid tissues and in PTC, with a progressive decline during neoplastic tumourigenesis, particularly in more aggressive histotypes. We present the first evidence of MR gene and protein expression in both normal and pathological thyroid cells and tissues. We have shown that MR is present and functionally activated in thyroid tissue. Binding of Aldo to MR induces the expression of inflammatory and thyroid-specific genes, and the thyroid may thus be considered a novel mineralocorticoid target tissue.

## 1. Introduction

Aldosterone (Aldo) is the main mineralocorticoid hormone produced by the adrenal glands. It regulates the body’s fluid and electrolyte balance by increasing renal reabsorption of sodium and renal excretion of potassium and hydrogen ions. In target cells, Aldo exerts its action through binding to the intracytoplasmic mineralocorticoid receptor (MR) encoded by *NR3C2* [1].

MR is part of the larger family of nuclear steroid hormone receptors. 11β-Hydroxysteroid dehydrogenase type 2 (11βHSD2) confers the specificity of mineralocorticoid action in target cells through the conversion of active cortisol into inactive cortisone. Aldo exerts its action through genomic (in hours–days) and non-genomic (in seconds–minutes) effects. The target cells of Aldo are mainly epithelial cells, particularly in the kidney, salivary glands, and gut [2]. However, MR is also located in non-epithelial cells, such as brain neurons [3], cardiomyocytes [4], vascular smooth muscle cells [5], endothelial cells [6], mononuclear leukocytes [7], pre-adipocytes and adipocytes [8,9] and erythrocytes [10]. In non-epithelial cells, Aldo induces inflammatory processes such as collagen formation, fibrosis, and necrosis, representing an important cardiovascular risk factor, regardless of its hypertensive effects [11]. Aldo also plays an immunostimulatory role, and MR expression has been demonstrated in cells involved in innate and adaptive immune responses, such as macrophages, dendritic cells (DCs), T lymphocytes, and naϊve B lymphocytes [12,13]. We previously demonstrated an increase in PAI-1 and p22(phox) expression, the main constituents of NADPH oxidase, in mononuclear leukocytes after incubation with Aldo and a decrease in the same proteins after co-incubation with canrenone (an MR antagonist), confirming the pro-oxidant action of mineralocorticoids [12]. Moreover, Carvajal and collaborators demonstrated that MR activation in response to Aldo stimulates the MAP kinase pathway in DCs by promoting transforming growth factor beta (TGF-β) and interleukin-6 (IL-6) secretions [14].

Furthermore, following Aldo stimulation, DCs activate CD8+ T lymphocytes and polarise the differentiation of CD4+ T helper lymphocytes towards a T helper 17 (Th-17) phenotype [15], which plays a role in promoting inflammation and autoimmune disorders, particularly in Hashimoto’s thyroiditis (HT) [16,17].

The relationship between primary aldosteronism (PA) and the development of thyroid disease has not been widely investigated, although some—rather preliminary—clinical evidence is reported in the literature. A significant increase in the prevalence of HT and non-toxic multinodular goitre has been reported in patients with PA compared with healthy normotensive controls matched for age, sex, iodine intake, and geographical area [18,19]. As further proof of the role of Aldo in stimulating the autoimmune response and the possible link with HT, Krysiak and colleagues in 2011 published a case report of a patient with concomitant HT and PA induced by Aldo-producing adrenal adenoma, whose surgical removal led to an improvement in the clinical course of HT (in terms of thyroid function and improvement in thyroid autoimmunity) [20].

Given these premises, the aim of this study was to investigate the possible gene and protein expression of MR in normal thyroid cells and in thyroid cancer cells, thereby elucidating the role of MR and mineralocorticoids in thyroid physiology and pathology.

## 2. Results

### 2.1. Gene Expression of Mineralocorticoid Receptor in Thyroid Tissues and Cell Lines

**Figure 1:**
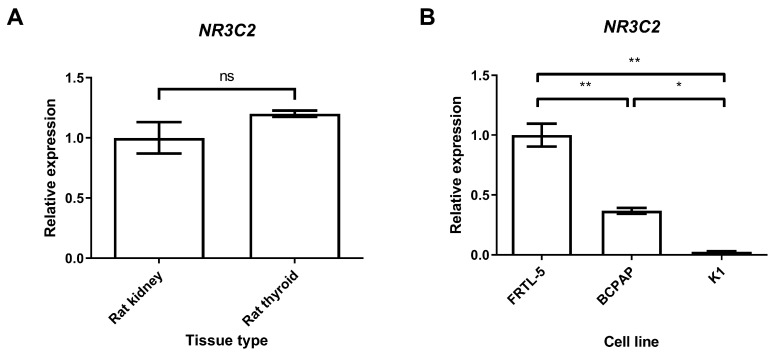
(**A**,**B**) *NR3C2* mRNA expression levels assessed by quantitative real-time polymerase chain reaction. (**A**) *NR3C2* expression analysis in different rat tissue types; (**B**) *NR3C2* mRNA expression levels in different cell lines. Experiments were performed in triplicate. ** = *p* < 0.01, * = *p* < 0.05.

Firstly, *NR3C2* expression in rat thyroid tissue was assessed by real-time quantitative PCR (qRT-PCR) using rat kidney tissue as the positive control. Our results revealed for the first time that the mRNA of MR is present in rat thyroid tissues and that its expression is comparable to that in rat kidney samples (Figure 1A).

MR was also found to be present in the Fischer rat thyroid cell line (FRTL-5) and in the two human papillary thyroid cancer (PTC) cell lines (BCPAP and K1), although it was reduced in the latter (0.37-fold in BCPAP and 0.03-fold in K1) compared with the former, used as the control (*p* < 0.01; Figure 1B), and in the K1 compared with the BCPAP cell line (*p* < 0.05; Figure 1B).

### 2.2. Mineralocorticoid Receptor Protein Expression, Localisation, and Activation in FRTL-5

**Figure 2:**
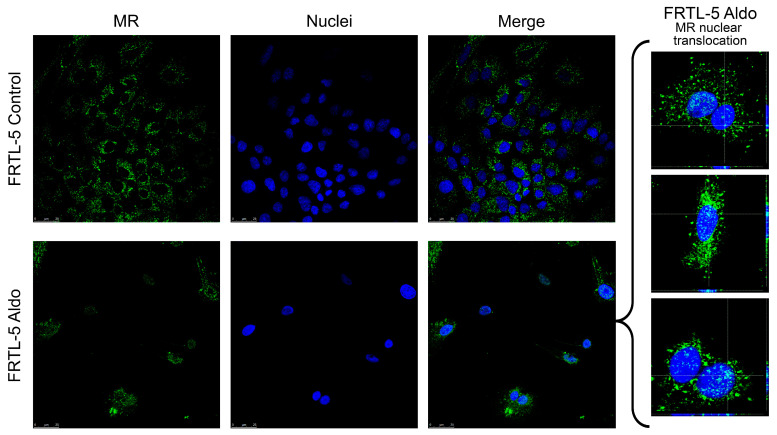
Confocal microscopy showing MR protein expression, localisation, and activation in the FRTL-5 cell line. Nuclear translocation of MR in FRTL-5 treated with aldosterone (1 µM for 24 h) can be seen in the expanded image detail. MR is shown in green; DNA stained with DRAQ5™ is shown in blue. Z-interval: 1 μm. Magnification: 63× oil immersion objective. Scale bar = 25 μm. MR = mineralocorticoid receptor.

Confocal microscopy was used to investigate the presence and localisation, of the MR protein in the FRTL-5 cell line. As Figure 2 shows, MR (in green) was found in the cytoplasm but was not detected in the nucleus of the control cells. After 24 hours’ stimulation with 1 µM Aldo, the receptor protein was observed to have increased in the proximity of the nucleus (marked in blue), and there was an evident translocation in the nucleus, as shown in the orthogonal projection reported in Figure 2.

### 2.3. Effects of Aldosterone on Target Genes Involved in Inflammation in the FRTL-5 Cell Line

**Figure 3:**
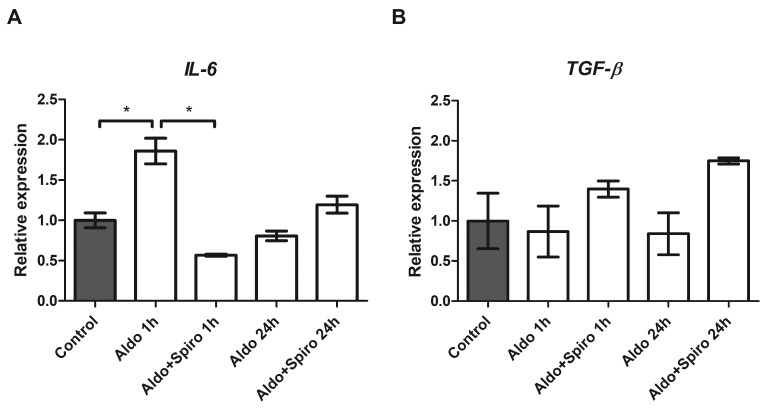
(**A**,**B**) mRNA expression levels in the FRTL-5 cell line assessed by quantitative real-time polymerase chain reaction. (**A**) IL-6 gene expression; (**B**) TGF-β gene expression. Aldo 1 h = Aldosterone 1 µM for 1 h; Aldo+Spiro 1 h = Aldosterone 1 µM combined with Spironolactone 10 µM for 1 h; Aldo 24 h = Aldosterone 1 µM for 24 h; Aldo+Spiro 24 h = Aldosterone 1 µM combined with Spironolactone 10 µM for 24 h. Experiments were performed in triplicate. * = *p* < 0.001.

We then investigated the effects on inflammation genes of treatment with Aldo alone or in combination with spironolactone (Spiro) at two different time points (after 1 h and after 24 h) in the FRTL-5 cell line. After 1 h, treatment with Aldo resulted in a 1.87-fold upregulation of IL-6 compared with the control (*p* = 0.0004) (Figure 3A). Notably, this effect was reversed when Aldo was co-incubated with Spiro (*p* < 0.0001). We also tested the effect on TGF-β expression but found no statistically significant differences after 1 h or after 24 h (Figure 3B).

### 2.4. Effects of Aldosterone on Thyroid-Specific Genes in the FRTL-5 Cell Line

We also investigated the effects on thyroid-specific gene expression of treatment with Aldo alone or in combination with Spiro at two different time points (after 1 h and after 24 h) in the FRTL-5 cell line.

**Figure 4:**
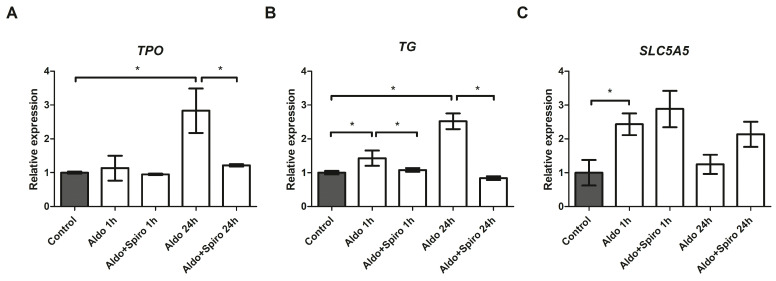
(**A–C**) mRNA expression levels in the FRTL-5 cell line assessed by quantitative real-time polymerase chain reaction. (**A**) Thyroid peroxidase gene expression levels; (**B**) Thyroglobulin gene expression levels; (**C**) *SLC5A5* (NIS) gene expression levels. Aldo 1 h = Aldosterone 1 µM for 1 h; Aldo+Spiro 1 h = Aldosterone 1 µM combined with Spironolactone 10 µM for 1 h; Aldo 24 h = Aldosterone 1 µM for 24 h; Aldo+Spiro 24 h = Aldosterone 1 µM combined with Spironolactone 10 µM for 24 h; TPO = thyroid peroxidase; TG = thyroglobulin. Experiments were performed in triplicate. * = *p <* 0.01.

After 24 h, treatment with Aldo resulted in a 2.83-fold upregulation of thyroid peroxidase (TPO; *p <* 0.001) and a 2.52-fold upregulation of thyroglobulin (TG; *p <* 0.001) mRNAs compared with the untreated control group (Figure 4A,B). In both cases, the effect was reversed when Aldo was co-incubated with Spiro (*p =* 0.0016 and *p <* 0.001, respectively). Furthermore, treatment with Aldo resulted in a 1.43-fold increase in TG expression after 1 h (*p =* 0.002), and again, the effect was reversed (*p =* 0.003) when Aldo was combined with Spiro.

We also tested the effects on *SLC5A5* (NIS) expression and found that treatment with Aldo (1 h) resulted in a 2.43-fold upregulation of NIS (*p =* 0.0014) (Figure 4C), an effect that was not reversed when combined with Spiro.

### 2.5. Mineralocorticoid Receptor Protein Expression, Localisation, and Activation in PTC Cell Lines

**Figure 5:**
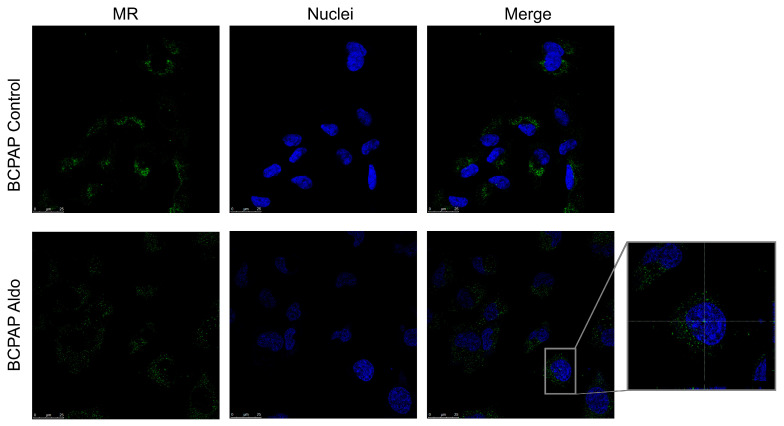
Confocal microscopy showing MR protein expression, localisation, and activation in BCPAP. Nuclear translocation of MR in BCPAP treated with aldosterone can be seen in the expanded image detail. MR is shown in green; DNA stained with DRAQ5™ is shown in blue. Z-interval 1 μm. Magnification: 63× oil immersion objective. Scale bar = 25 μm. MR = mineralocorticoid receptor.

Confocal microscopy was used to investigate the presence and localisation of MR in two different PTC cell lines. Figure 5 shows that MR (in green) was found at the cytoplasmatic level in the BCPAP but was not detected in the nucleus (marked in blue) at baseline. After 24 h stimulation with Aldo 1 µM, a modest translocation in the nucleus was observed.

**Figure 6:**
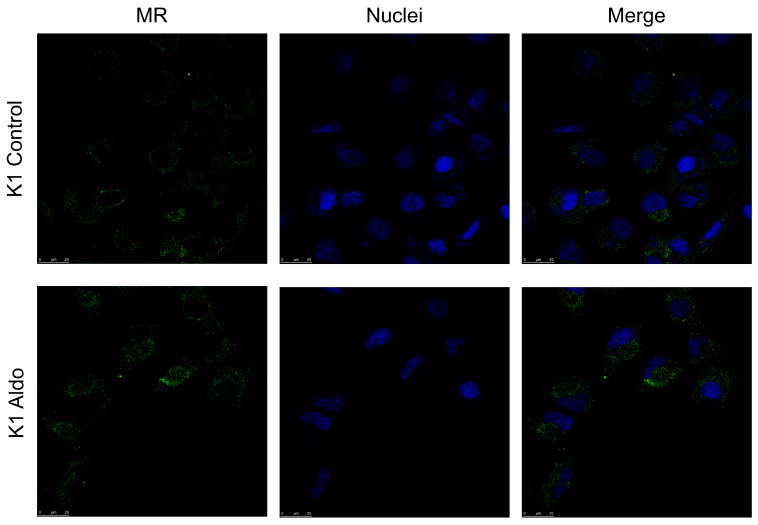
Confocal microscopy showing MR protein expression, localisation, and activation in K1. The mineralocorticoid receptor is shown in green; DNA stained with DRAQ5™ is shown in blue. Z-interval: 1 μm. Magnification: 63× oil immersion objective. Scale bar = 25 μm. MR = mineralocorticoid receptor.

MR was also present in the cytoplasm of the K1 cell line at baseline, but, unlike the BCPAP cell line, nuclear translocation was not detected following treatment with Aldo (Figure 6).

### 2.6. Effects of Aldosterone in PTC Cell Lines

**Figure 7:**
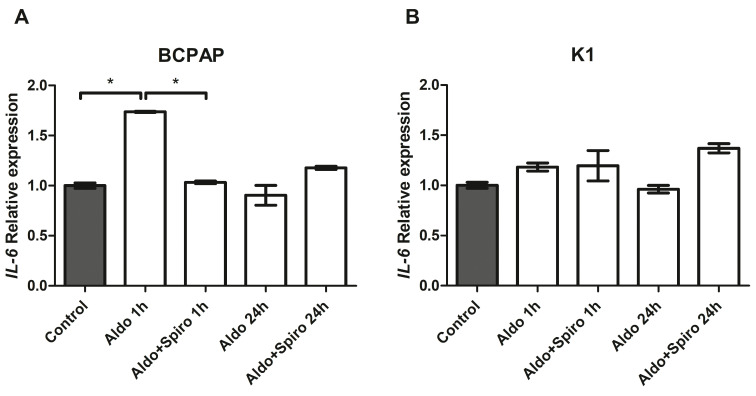
(**A**,**B**) IL-6 mRNA expression levels in the PTC (BCPAP and K1) cell lines assessed by quantitative real-time polymerase chain reaction. (**A**) IL-6 gene expression in the BCPAP cell line; (**B**) IL-6 gene expression in the K1 cell line. Aldo 1 h = Aldosterone 1 µM for 1 h; Aldo+Spiro 1 h = Aldosterone 1 µM combined with Spironolactone 10 µM for 1 h; Aldo 24 h = Aldosterone 1 µM for 24 h; Aldo+Spiro 24 h = Aldosterone 1 µM combined with Spironolactone 10 µM for 24 h. Experiments were performed in triplicate. * = *p <* 0.001.

We investigated the effects on inflammation genes of Aldo treatment alone or combined with Spiro at two different time points (after 1 h and after 24 h) in the two PTC cell lines. In BCPAP cells, treatment with Aldo for 1 h resulted in a 1.73-fold upregulation of IL-6 gene expression (*p <* 0.001) compared with the control (Figure 7A). Again, this effect was reversed when Aldo was combined with Spiro (*p <* 0.001). In contrast, at neither time point did treatment with Aldo induce any increase in IL-6 expression in the K1 cell line (Figure 7B).

We also tested the effect on TGF-β gene expression and observed no significant effects in either cell line or at either time point.

Investigation of the effect on thyroid-specific genes revealed no statistically significant effect on *SLC5A5* (NIS) gene expression in BCPAP cells at either time point, and no *SLC5A5* expression was detected in the K1 cell line.

Finally, no TPO or TG gene expression was detected in either cell line.

### 2.7. Mineralocorticoid Receptor Gene and Protein Expression in Human PTC

**Figure 8:**
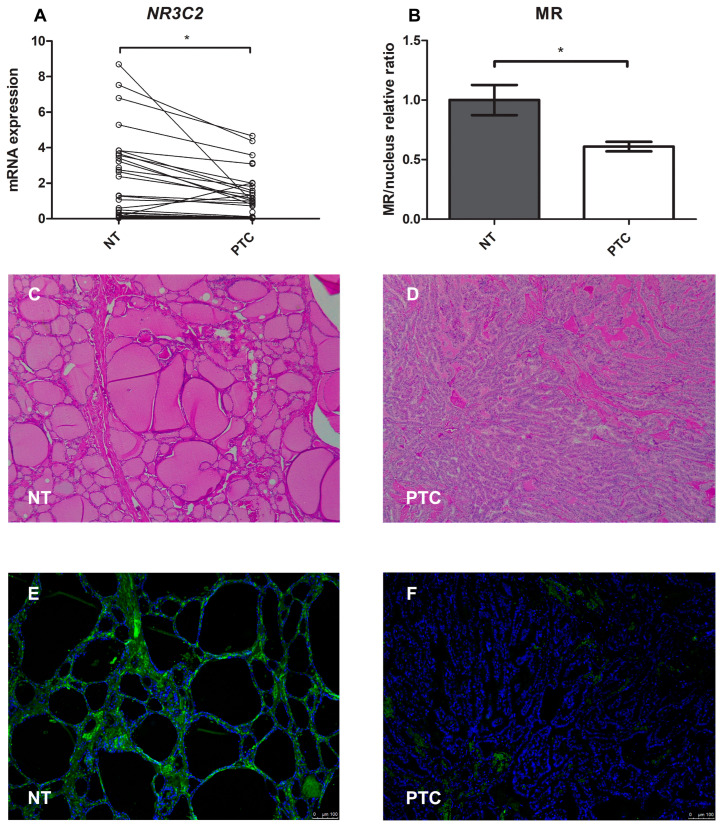
(**A**–**F**) Mineralocorticoid receptor gene and protein expression in normal human thyroid tissue compared with papillary thyroid cancer tissue. (**A**) Representation of the Wilcoxon test for paired data on *NR3C2* mRNA expression: each PTC tissue sample was compared with its healthy tissue counterpart; (**B**) Quantification of mean fluorescence intensity (MFI); (**C**,**D**) Representative images of the immunohistochemistry analysis of normal thyroid tissue and papillary thyroid cancer tissue stained with hematoxylin and eosin; (**E**,**F**) Immunofluorescence of normal thyroid tissue and papillary thyroid cancer tissue (green = MR, blue = nucleus). Magnification: objective 10×. Scale bar = 100 μm. NT = normal thyroid tissue; PTC = papillary thyroid cancer. * = *p <* 0.001.

We also analysed and compared *NR3C2* gene expression in cancer and normal thyroid tissue from the same patient (a total of 29 patients). First of all, we found significant MR gene expression in both the PTC and normal thyroid tissue. However, there was a significant reduction in *NR3C2* mRNA expression levels in the neoplastic compared with the normal thyroid tissue, as shown in Figure 8A (median 0.46 and 0.58, respectively, *p <* 0.001).

For the immunofluorescence analysis, the MR protein was marked with green and the nucleus with blue (Figure 8E,F). Quantification of the mean fluorescence intensity (MFI) showed that MR protein expression was lower (0.61-fold, *p <* 0.001) in pathological tissue than in healthy tissue, as shown in Figure 8B.

**Figure 9:**
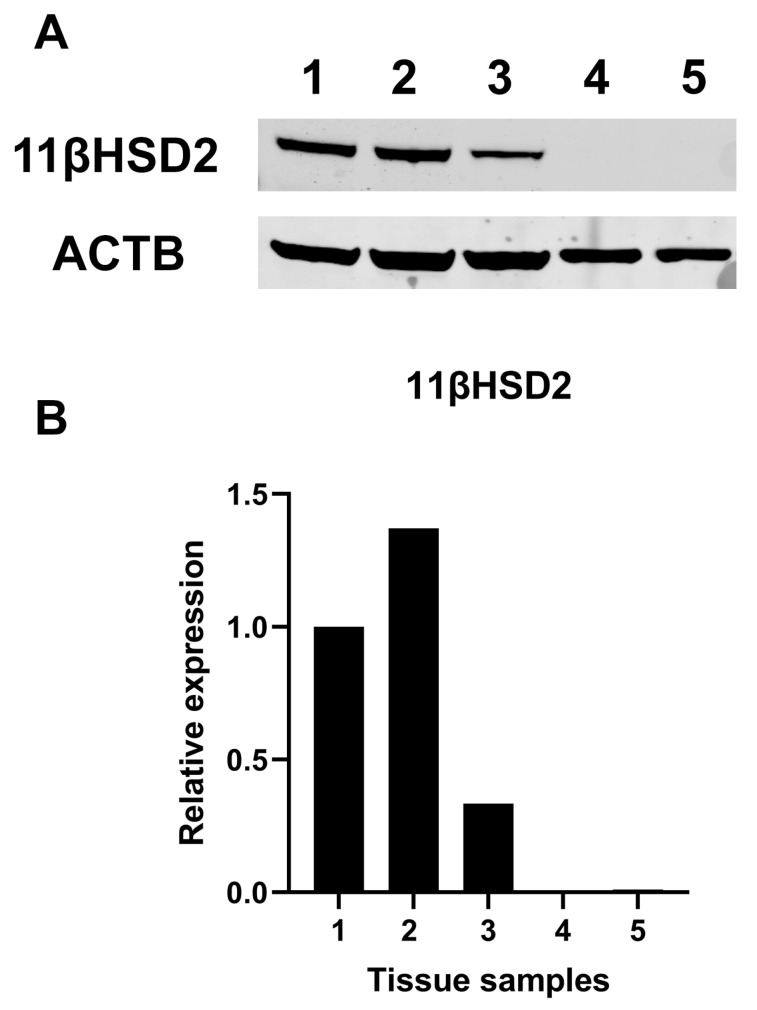
Western blot analysis for 11β-Hydroxysteroid dehydrogenase type 2. (**A**) Representative Western blot analysis for different tissue samples. (**B**) Quantification of band intensity, Samples 1–3: healthy counterparts of human kidney carcinoma specimens; Samples 4, 5: healthy counterparts of human papillary thyroid cancer. 11βHSD2 = 11β-Hydroxysteroid dehydrogenase type 2.

Finally, we did not find significant protein expression of 11βHSD2 in normal human thyroid tissues in contrast to human kidney tissues (Figure 9).

### 2.8. Mineralocorticoid Receptor Gene Expression in Aggressive Thyroid Cancers

We collected 47 classic variants of PTC, 9 hobnail variants of PTC, 3 anaplastic thyroid cancers (ATC), and 10 medullary thyroid cancers (MTC) to evaluate the differences in *NR3C2* gene expression among aggressive thyroid cancers.

**Figure 10:**
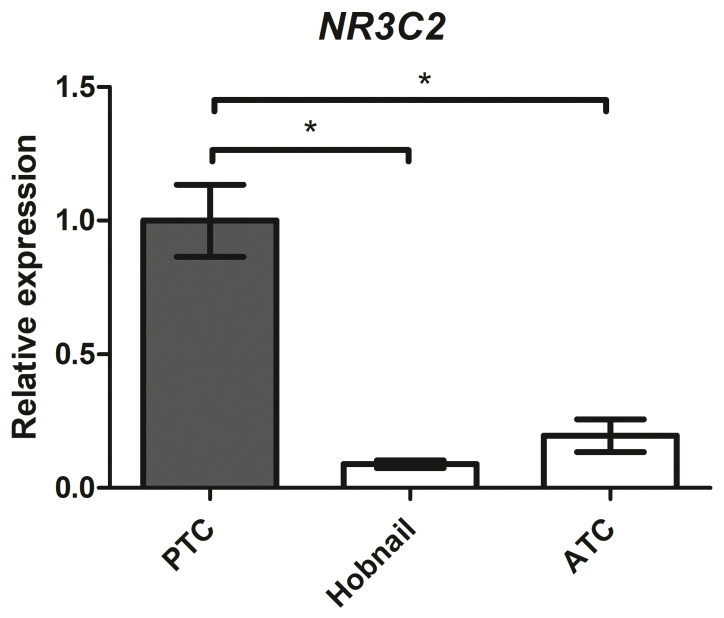
*NR3C2* mRNA expression levels in different human thyroid cancer tissues assessed by quantitative real-time polymerase chain reaction. PTC = classic variant of papillary thyroid cancer; ATC = anaplastic thyroid cancer. * = *p <* 0.001.

MR expression levels were significantly reduced in the more aggressive thyroid histotypes (Figure 10): 0.09-fold in the hobnail variant (*p <* 0.001) and 0.20-fold in ATC (*p <* 0.001) compared with the classic variant of PTC. No MR gene expression was detected in the 10 MTC patients.

## 3. Discussion

Through binding with MR, Aldo classically controls fluid and electrolyte homeostasis in the MR-sensitive distal nephron. However, decades of research have demonstrated MR expression in other non-classical target tissues as well, such as heart muscle cells, endothelial cells, smooth muscle cells, and inflammatory cells (T cells, macrophages, and DC) [21]. In these tissues, MR plays a role in activating the inflammatory process by stimulating the oxidative pathway, provoking cell activation, and increasing the production of pro-inflammatory and pro-fibrotic mediators [22,23]. To date, the thyroid has not been known to belong to the non-classical target tissues of mineralocorticoids.

This is the first time, to our knowledge, that MR has been demonstrated to be present in normal and pathological thyroid cells in both rats and humans.

The MR has a similar affinity for Aldo and cortisol. The presence of 11βHSD2 in the classical target tissues protects the receptor from binding with cortisol, which is inactivated by cortisone. Many studies have shown that MR is also present in other tissues such as cardiomyocytes, endothelial cells, inflammatory cells, and mononuclear leukocytes, and in these tissues that do not possess 11βHSD2, the main ligand is cortisol [4,6,7]. However, in situations of inflammation, Aldo can play a direct role in these tissues as well. As an example, the cardiomyocytes normally do not express 11βHSD2 [24,25]. Therefore, it is widely accepted that glucocorticoids normally occupy MR in cardiac tissue rather than Aldo. However, under pathological conditions, such as hypoxia, 11βHSD2 is also overexpressed in this tissue, and Aldo may compete for binding to the MR [26]. In heart tissue, Aldo contributes to ventricular remodelling after acute myocardial infarction, and MR antagonists exert beneficial effects, reducing sudden cardiac death and hospitalisations for heart failure when used early after myocardial infarction [27,28]. Although there is no protein expression of 11βHSD2 in thyroid tissue under physiological conditions, we could speculate that in thyroid diseases 11βHSD2 may be overexpressed too.

Our data clearly demonstrated that MR is present, functionally recruitable, and associated with an increased expression of inflammatory and thyroid-specific genes under Aldo stimulation. Consequently, we may consider the thyroid as a novel, non-classical target tissue for adrenal cortex hormones.

Initially, we found MR gene expression in both renal and thyroid rat tissues at comparable levels, suggesting an unexpectedly strong presence of MR in the thyroid. Then we chose FRTL-5 as our normal rat thyroid cells in our in vitro model and not only confirmed MR gene expression but also showed protein expression and receptor activation (nuclear translocation) after stimulation with its physiological agonist (Aldo). Moreover, we took two human PTC cell models, the BCPAP and K1 cell lines, and here, too, we demonstrated MR gene and protein expression, although to a lesser extent, and also observed differences between the two cell lines, with MR gene expression being 10-fold higher in BCPAP than in K1. This difference can in all likelihood be attributed to their intrinsic differences, as the BCPAP cell line derives from a poorly differentiated PTC and the K1 cell line derives from the metastasis of a well-differentiated PTC [29]. In addition, BCPAP carries the *BRAF* (V600E) and *TP53* mutations (D259Y), while the K1 line carries the *BRAF* (V600E) and *PI3KCA* mutations (E542K) [30].

As is already known, MR activation in non-classical tissues has pathological effects such as excessive extracellular matrix accumulation, oxidative stress, and prolonged inflammation [31]. Therefore, we sought to investigate the possible pro-inflammatory effects of MR in the thyroid by assessing IL-6 and TGF-β Aldo-induced gene expression in the cell lines. We found a strong, acute increase only in IL-6 expression, but not in TGF-β, in both FRTL-5 and the BCPAP lines compared with controls. The effect of Aldo was negated by co-incubation with Spiro in both cell lines.

Our seminal in vitro findings could represent the molecular basis for the only supposed link between PA and thyroid diseases—such as HT and multinodular goitre—in vivo. In fact, a few studies have demonstrated this epidemiological association [18,19,32]. Interestingly, Turchi and colleagues found that at ultrasonography, the prevalence of thyroid abnormalities was higher in PA patients than in essential hypertensive patients (66% vs. 46%) [32]. In particular, 47% of the patients in their study had multinodular goitres, 16% had solitary nodules, and 3% had PTC. We can speculate that MR activation in the thyroid promotes chronic inflammatory processes (e.g., IL-6-mediated processes), leading to thyroid alterations.

We also investigated whether MR pathways were involved in modulating the key thyroid-specific genes, such as TG, TPO, and NIS. Interestingly, after Aldo treatment in the FRTL-5 cell line, we observed acute (after 1 h) and late (after 24 h) TPO upregulation, the latter antagonised by the addition of Spiro; acute and late TG upregulation, both antagonised by Spiro; and only acute NIS upregulation. These results show that MR activation may be involved in overexpression of the main thyroid-specific genes. What the role of this new signalling connecting mineralocorticoids and thyroid might be under physiological or pathological conditions is currently unknown and a matter for further studies. Remarkably, previous reports have shown that in mouse kidney models, Aldo and his analog deoxycorticosterone pivalate (DOCP) were able to promote upregulation at both the mRNA and protein levels of Pendrin, an anion exchanger protein expressed not only along the apical plasma membrane of intercalated cells of the distal convoluted tubule, connecting tubule, and cortical collecting duct in the kidney, where it plays a role in acid/base balance, but also along the apical membrane of thyrocytes, where it is involved in mediating iodide efflux [33,34]. Basically, our in vitro model—the FRTL-5 cell line—does not express pendrin, so we could not evaluate its expression in the case of MR activation by Aldo.

The increased expression of TGF-β and NIS that is evident in the FRTL5 cell line after the addition of Spiro may be explained considering that Spiro is a drug itself with possible cross-reactivity with other receptors not related to the mineralocorticoid pathway.

The prevalence of PTC in PA is unclear, although a few studies and case reports have drawn attention to a possible association between PA and PTC [35,36,37], given that PTC has a prevalence in the range of 3–18.5% in PA patients [32,37]. In light of these observations, we wanted to assess the gene and protein expression of MR in human thyroid tissues. To do so, we collected a series of paired samples of normal and neoplastic thyroid from patients diagnosed with PTC and obtained the first proof of MR gene and protein expression in both normal human thyroid tissue and PTC. As expected, we found a significant reduction in MR expression at both the gene and protein levels in the cancer sample compared with the normal sample of each patient. We also analysed MR gene expression in tissues of more aggressive histotypes of thyroid cancer—hobnail variants of PTC and ATC—and found it to be significantly reduced in these variants compared to the classic variant of PTC. The role of dedifferentiation in cancer cell heterogeneity, initiation, and progression is widely accepted [38]. We can speculate that the reduction in MR expression may be an indication of cellular dedifferentiation, typical of the carcinogenic process. Alternatively, the loss of MR expression may give the thyroid cancer cells a replicative advantage, which finally leads to the selection of the clone. In any case, the loss of mineralocorticoid signaling confers an advantage to thyroid neoplastic cells, raising speculation about the possible antiproliferative role of Aldo or MR agonists that will need to be demonstrated in future studies.

The discovery of MR in the thyroid represents a first step in elucidating a potential pathophysiological role for Aldo in the thyroid. However, further studies are needed to verify our findings and clarify the MR function in the thyroid.

## 4. Materials and Method

### 4.1. Rat and Human Tissues

A tissue bank has been in operation at the University Hospital of Padua since 2005. Patients about to undergo surgery for certain diseases (including thyroid cancer) are routinely asked for permission to collect and store their tissue and other samples for research purposes (protocol ref. 3388). All patients involved in this study gave written informed consent to the banking of their tissue samples, with the approval of the ethics committee of Padua Hospital [39]. The study was conducted in accordance with the Declaration of Helsinki.

We examined a series of different thyroid tissue samples: 47 classic variants of PTC, 9 hobnail variants of PTC, 3 ATC, and 10 MTC.

Twenty-nine of the 47 patients diagnosed with the PTC classic variant also presented with the healthy counterpart. We conducted qPCR analyses to study the gene expression of human *NR3C2*.

The group of PTC patients comprised 15 males and 32 females and had a mean age of 46 ± 15 years.

The rat kidney and thyroid cDNA were kindly provided by Marnie Granzotto (University of Padua). RNA was extracted from the thyroid and kidney tissue of a Sprague-Dawley rat that was used as a donor by the Zymo DirectZol RNA Miniprep kit (Zymo Research, cat. no. R2052, Irvine, CA, USA) according to the manufacturer’s instructions. cDNA synthesis was performed with the High Capacity cDNA Reverse Transcription Kit (Applied Biosystems by Thermo Fisher Scientific, Monza, Italy).

### 4.2. Cell Cultures and Maintenance

The FRTL-5 (RRID:CVCL_0265) was obtained from CLS (Cell Lines Service GmbH, Eppelheim, Germany). It was cultured in Ham’s F12 Coon’s modification cell growth medium supplemented with TSH (final concentration 16.5 ng/mL), L-glutamine, and FBS (Cell Lines Service GmbH, Eppelheim, Germany).

The BCPAP (human PTC cell line, RRID:CVCL_0153) was obtained from the German Collection of Microorganisms and Cell Cultures (Leibniz Institute DSMZ, Braunschweig, Germany); the K1 (human PTC cell line, RRID:CVCL_2537) was obtained from the European Collection of Authenticated Cell Cultures (ECACC, Sigma-Aldrich S.r.l., Milan, Italy).

The BCPAP and K1 cell lines were cultured in RPMI 1640 (Gibco, Life Technologies, Carlsbad, CA, USA) supplemented with 10% foetal bovine serum (FBS) (Gibco), L-glutamine (2 mM), and penicillin/streptomycin (100 IU/mL and 100 μg/mL, respectively).

Adherent monolayer cultures were maintained in T75 culture flasks and incubated at 37 °C with 5% CO_2_ until they reached 85% confluency. Cells were detached using 0.025% trypsin (Sigma-Aldrich) and plated into T75 flasks at a density of 2 × 10^6^ cells.

### 4.3. Drugs

Aldo (cat. no: A9477) and Spiro (cat. no: S3378) were purchased from MERCK.

The powders were dissolved in a 10 mM stock solution of dimethyl sulfoxide (DMSO) and stored at −80 °C. In our study, we chose a concentration of 1 µM of Aldo because in other similar studies on different cell cultures, the same dose was used [40,41].

### 4.4. RNA Extraction, cDNA Synthesis, and qRT-PCR Analysis

Total RNA was extracted using TRIzol reagent as the lysis buffer (Invitrogen, Carlsbad, CA, USA) according to the manufacturer’s protocol, and qRT-PCR was performed with a QuantStudio™ 5 Real-Time PCR System (Applied Biosystems, Milan, Italy) and the relative quantification (2^−ΔΔCT^) method, as described elsewhere [39]. The genes were analysed using the TaqMan assays shown in Table 1, all from Applied Biosystems. The reference genes used in this study were human ACTB (Hs99999903_m1) and rat ACTB (4331182; Rn00667869_m1), the latter one of the most frequently used housekeeping genes [42].

### 4.5. Immunofluorescence and Confocal Microscopy

The cells were seeded at appropriate concentrations in 12-well plates with a polylysine-coated coverslip. The following day, the cells were treated with Aldo, and 24 h later, they were fixed in 2% PFA (Cat. No. A11313-22, Thermo Fisher, Monza, Italy) for 20 min, then permeabilised with 0.5% Triton X-100 in PBS for 15 min. All steps were performed at room temperature.

Primary and secondary antibodies were diluted in PBS containing 0.5% BSA.

MR was visualised using a 1:100-diluted NR3C2 mouse monoclonal antibody (H10E4C9F; Thermo Fisher Scientific; Cat. No. MA1-620, RRID:AB_2298880), followed by a 1:300-diluted goat anti-mouse IgG polyclonal antibody, FITC conjugated (Sigma-Aldrich, Cat. No. F0257, RRID:AB_259378) for confocal microscopy.

Secondary antibodies were also used in the absence of primary antibodies to assess non-specific binding. For the analysis, DNA was stained using a DRAQ5™ Fluorescent Probe (Thermo Fisher Scientific, Cat. No. 62252) at 1:300 in PBS for 30 min at 37 °C. Finally, the cells were mounted with Mowiol anti-fade solution (Sigma-Aldrich).

Cell samples (FRTL-5, BCPAP, and K1) were analysed by confocal microscopy (Leica TCS SP8, Leica Microsystems) with a z-range of 1 μm using a 63×/1.4 oil immersion objective (image size 1024 × 1024 pixels).

Paraffin-embedded sections of thyroid tissue were provided by the Surgical Pathology Unit of the University Hospital of Padua. Slides were dewaxed and rehydrated through xylene (3 × 20 min) and graded alcohols (100%, 95%, 70%; 5 min each).

The slides were permeabilised with 0.5% Triton X-100 in PBS for 20 min and washed with PBS. MR was visualised as described before.

The slides of the patients’ tissue samples were viewed under a Leica DMI6000CS fluorescence microscope (Leica Microsystems, Buccinasco, Milan, Italy) and analysed with differential interference contrast (DIC) and fluorescence objectives. Images were acquired with a 10 × 0.25 dry objective (image size 1024 × 1024 pixels).

All images were captured using a DFC365FX camera and processed with the Leica Application Suite (LAS-AF) 3.1.1. software (Leica Microsystems, Buccinasco, Milan, Italy).

### 4.6. Western Blot Analysis of Human Tissues

Normal human renal and thyroid tissues were homogenized with the Qiagen Tissue Lyser System (QIAGEN, Courtaboeuf, France) and immediately frozen in liquid nitrogen. Then proteins were extracted using RIPA buffer.

Proteins were separated with SDS/PAGE under reducing conditions in the presence of the S-S reducing agent dithiothreitol, then electroblotted onto nitrocellulose membranes and saturated in dry 5% non-fat milk. Membranes were incubated overnight with the primary antibodies (anti-11βHSD2 ABclonal Cat# A8077, RRID:AB_2769875, diluted 1:500, and anti-β-actin ABclonal Cat# AC004, RRID:AB_2737399, diluted 1:50,000).

Primary antibodies were detected with two secondary fluorescent antibodies, an anti-mouse (1:5000, IRDye 680RD) and an anti-rabbit (1:10,000, IRDye 800CW), both from Li-Cor Biosciences, Milan, Italy. Membranes were scanned using the LI-COR Odyssey CLx Imaging System, and the band intensity was quantified with the LI-COR Empiria Studio^®^ Software (v.2.1). Signals were normalised on the ACTB signal and presented as expression relative to a normal human renal specimen (sample 1).

### 4.7. Statistical Analysis

All statistical analyses were performed with the MedCalc^®^ Statistical Software (MedCalc Software Ltd., 2022, version 22.017, Ostend, Belgium). The Kolmogorov–Smirnov test was used to assess the normal distribution of all variables. All data were expressed as means ± standard deviations (SD) for normally distributed variables and as medians and interquartile ranges (IQR) for non-normally distributed variables.

The Mann–Whitney test, Kruskal–Wallis test (with Conover’s post hoc analysis), Student’s *t* test, and Wilcoxon test for paired data were used, as appropriate, to compare: *NR3C2* mRNA expression levels in different rat tissue types and in the FRTL-5 cell line vs. the PTC cell lines; the effects of Aldo on target genes involved in inflammation and thyroid-specific genes in the FRTL-5 cell line; the effects of Aldo on different PTC cell lines; MR gene and protein expression in human PTC tissue vs. normal thyroid tissue and in different variants of PTC.

All results were considered statistically significant at *p* < 0.05.

## 5. Conclusions

We have produced the first evidence in the literature of MR gene and protein expression in both normal and tumoral thyroid cells and tissues, identifying the thyroid and thyroid-related genes as new possible targets for mineralocorticoid action. We have thus revealed a potential molecular substrate for the assumed link between PA and thyroid diseases. We are confident that in the future, a better understanding of the crosstalk between Aldo and thyroid tissue could offer new insights into the pathogenesis of benign and malignant thyroid disease and provide new molecular targets, especially in thyroid cancer treatment.

## Figures and Tables

**Table 1:** List of the TaqMan probes used in the qPCR assays, with their gene names and symbols.

Assay ID	Gene	Gene Symbol	Cat. No.
Hs01031809_m1	Human Mineralocorticoid Receptor (MR)	*NR3C2*	4331182
Hs001741341_m1	Human Interluekin 6	*IL-6*	4331182
Hs00998133_m1	Human Transforming Growth Factor-β	*TGFβ*	4331182
Hs00892519_m1	Human Thyroid Peroxidase	*TPO*	4331182
Hs00174974_m1	Human Thyreoglobulin	*TG*	4331182
Hs00166567_m1	Human Sodium/Iodide symporter (NIS)	*SLC5A5*	4331182
Hs00388669_m1	Human Hydroxysteroid 11-β dehydrogenase 2	*HSD11B2*	4331182
Rn00565562_m1	Rat Mineralocorticoid Receptor (MR)	*NR3C2*	4448892
Rn01410330_m1	Rat Interluekin 6	*IL-6*	4448892
Rn00572010_m1	Rat Transforming Growth Factor-β	*TGFβ*	4448892
Rn00571159_m1	Rat Thyroid Peroxidase	*TPO*	4448892
Rn00667257_g1	Rat Thyreoglobulin	*TG*	4448892
Rn00583900_m1	Rat Sodium/Iodide Symporter (NIS)	*SLC5A5*	4448892
Rn00492539_m1	Rat Hydroxysteroid 11-β Dehydrogenase 2	*HSD11B2*	4448892

## Data Availability

Data are available on request due to privacy restrictions.
